# Monthly Identification of High Frequency Emergency Presenters to Improve Care Delivery and Evaluation: A Unique Methodological Approach

**DOI:** 10.31486/toj.22.0080

**Published:** 2022

**Authors:** Andy Wong, Amy N. B. Johnston, Shane Collett, Robert Eley

**Affiliations:** ^1^ Department of Emergency Medicine, Princess Alexandra Hospital, Woolloongabba, Queensland, Australia; ^2^ School of Mechanical, Medical and Process Engineering, Queensland University of Technology, Brisbane, Queensland, Australia; ^3^ Faculty of Medicine, University of Queensland, St Lucia, Queensland, Australia

**Keywords:** *Emergency service–hospital*, *epidemiology*, *evaluation studies as topic*

## Abstract

**Background:** Frequent presenters to emergency departments (EDs) pose many challenges around care delivery and health service management. The aim of this study was to investigate the presentation patterns of people with 5 or more ED visits in any calendar month (5+ frequent presenter [FP5+]) to develop a useful methodological framework on which the real impact of interventions may be assessed.

**Methods:** This study is a retrospective analysis of de-identified frequent ED presentation data using segmented regression analysis of an interrupted time series (ITS).

**Results:** A total of 82 FP5+ to this single ED were identified in a year. Of these presenters, 77% had 10 or more presentations in a year. The total FP5+ presentations in the 12 months preceding and after each participant's ≥5 presentations in 1 month (the *trigger month* for inclusion in the study) accounted for 1,064 and 1,606 visits, respectively. ITS analysis of frequent ED presentations did not show a significant level change or trend change during the data collection period. Monthly review of people who frequently present to a single ED showed that presentations typically occurred in bouts that may span calendar years. Presentation bouts then typically slow, potentially distorting evaluation of the effects of interventions.

**Conclusion:** Rolling monthly examination of presentation data may facilitate timely case review and care delivery, as well as provide a holistic picture of the impacts of interventions targeting patient care needs. This unique analysis demands a reconsideration of the typical before-and-after analysis of interventions for this vulnerable and high-cost group of patients.

## INTRODUCTION

Capacities of emergency departments (EDs) to provide quality care is increasingly challenged by many factors, including increases in the absolute number and the percentage of people presenting for care and management.^1^ In Australia, 2020-2021 data suggest an increase in presentations of 6.9%,^2^ up from 3.4% in 2017-2018,^3^ reflecting worldwide trends.^4^ Increased presentations with relatively inelastic service delivery typically result in ED crowding. Crowding can compromise staff morale, patient outcomes, and ongoing health service capacity to deliver care.^5^ Internationally, policies and research foci are targeting reducing the demand for ED services.^4^

One area of focus is the small number of patients who present frequently to EDs.^6-10^ This group of patients, although small in number and proportion, is often associated with some of the poorest social determinants of health.^3,6,11-13^ These patients are the focus of many health studies, not just because of their complex care needs and demands, but also because economic modeling indicates that their repeated presentations to EDs are associated with significant costs that do not link to definitive care delivery.^7,14,15^ Patients who frequently access EDs are also more likely to frequently access other primary care services, with associated costs to those health services as well.^6,9,10,16-18^

Many interventions described in the literature aim to decrease the number and frequency of presentations by users with complex care needs and thereby reduce the service demands and financial impacts to EDs of managing these patients.^14,19,20^ Interventions include processes such as the development and implementation of individual, personalized case management.^11^ However, a lack of consistency in outcome measures and even in the definition of what constitutes a *frequent presenter* have hampered interpretation and comparison of study outcomes.^5,19,21^ Challenges in interpretation and comparison of study findings are compounded by a limited capacity to collect reliable data because of the relative inability to track patient presentations across health districts or health services.^20^ Presentations are typically collated by year—calendar year or 12-month block—in a single facility and analyzed using a range of statistical procedures.^19^ While such an approach may be useful to count the frequency of frequent ED presenters each year (or other time block), if presentations are not evenly (linearly) spaced, this approach may confound the development of baseline data. Adopting this approach to data collation may also compromise researchers’ capacity to accurately demonstrate impacts of altered models of care. Moreover, processes that require a minimum of 12 months to identify at-risk patients are unfeasible and clinically unjustifiable, particularly given the evidence that early rather than late intervention for those in need is likely to be more effective.^22,23^

Many researchers have called for a standardized definition of frequent presenters to enable early recognition and the delivery and evaluation of timely, targeted care.^19,21^ Thus, the aims of this study were (1) to investigate the presentation pattern of people who had 5 or more ED visits in a calendar month for possible implementation as a standardized definition of frequent presenters, and (2) to use these data to develop a validated methodology for future assessment and evaluation of interventions for frequent ED presenters. Such a methodology would necessarily consider the baseline pattern of presentations using a statistically sound analysis process.^24^ The frequent presenters’ dataset at the study site was used to explore evaluation techniques.

## METHODS

This study is a retrospective analysis of baseline frequent ED presentations during a 5-year period, using a novel analytical methodology that is known to accurately capture patterns of presentation.^25^

### Setting

The setting for this study was a large, inner-city, tertiary ED in Australia with more than 60,000 primary adult presentations per year for all specialties excluding obstetrics and gynecology.

### Context

In 2014, the Frequent Emergency Presenter Project was established at this hospital with the aim of developing a dataset of frequent presenters to inform the development of processes within the ED framework to identify, understand, and improve care delivery to and management of this group of patients. Monthly reports of ED presentations enabled identification of patients who had presented 5 or more times in a calendar month. In the absence of a widely accepted definition of frequent presenter,^5^ the choice of ≥5 presentations was based on existing literature and practical management considerations, and these patients were designated as a 5+ frequent presenter (FP5+). Each FP5+ was included in the Frequent Emergency Presenter Project database, triggering consideration of these cases by the Frequent Emergency Presenter Project team. After an initial screening process, a comprehensive summary of all medical, surgical, psychiatric, psychological, and social issues for each eligible patient was generated. This comprehensive patient review also included direct consultant-to-consultant communication about each specific patient's needs and clarification of the overarching plan from the specialist unit perspective to best identify, meet, and support each individual's health care needs when they presented to the ED.

### Data Source

Data for the study were drawn retrospectively from the electronic health records available for each included patient between 2011 and 2016. Based on their medical record number (known locally as UR or unique record), patients were selected if they had ≥5 presentations in any calendar month during the study period.

Every patient is given a UR when first registered at the hospital. For those revisiting the hospital, a well-established protocol identifies the previously assigned UR with the patient's name and date of birth. This process ensures that all medical files with the same UR are linked to the same unique person. Identified FP5+ patients and their average monthly presentations were recorded and analyzed within rolling 12-month periods (eg, January 2012 to December 2012, February 2012 to January 2013). Each record, reflective of an individual presentation, was assigned a unique analysis number to ensure later de-identification for reporting purposes. A single outlier, who recorded the highest yearly presentations (177) in 2013, was excluded because the individual had more than double the number of presentations of the patient with the second highest number of presentations.

### Data Analysis

To avoid the common limitations of uncontrolled studies,^24^ interrupted time series (ITS) analysis was applied in assessing the change in the average yearly presentations using the statistical package R, version 3.6.1 (The R Foundation), on the standard ITS model.^25^ The residuals of the ITS model were assessed by examining the plot of autocorrelation and by the Shapiro-Wilk test for normality.

### Ethical Approval

This study was conducted according to National Health and Medical Research Council guidelines and received ethics approval from the Health Service Human Research Ethics Committee (HREC/18/2021/323).

## RESULTS

**Figure 1.**
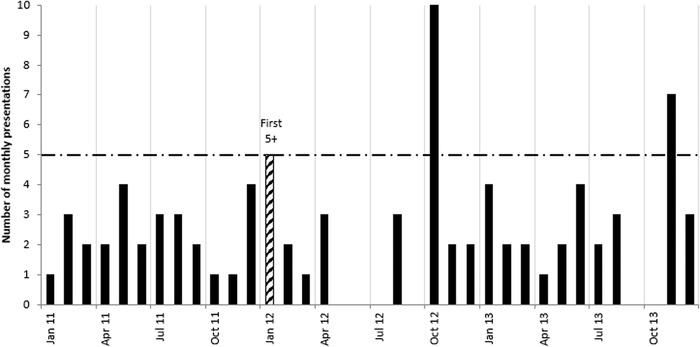
**Exemplar monthly presentation pattern of a frequent emergency department presenter across a 3-year period in the absence of any targeted intervention. Data include the year prior to and 2 years after the patient first presented ≥5 times per month**. Jan, January; Apr, April; Jul, July; Oct, October.

Figure 1 shows the monthly presentation pattern of a representative FP5+ during a 3-year period. The figure illustrates that the number of monthly ED presentations for an individual can vary, with sudden increases, declines, and then increases again in the absence of any targeted intervention.

**Figure 2.**
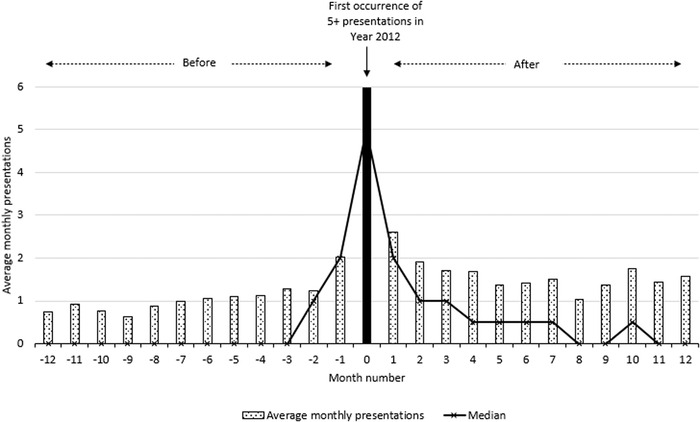
Average and median monthly presentations before and after the trigger month (the first documented instance of a patient presenting to the emergency department ≥5 times in a single calendar month). The horizontal axis represents a variable 12-month period before and after the trigger month in 2012. Thus, the data collected for presentations before and after the trigger month spanned the years 2011 and 2013, n=82.

Of the 82 FP5+ patients identified in 2012, 92% (75) had 7 or more presentations that year, while 77% (63) had 10 or more presentations to this single ED. When presentation data were plotted for the 12 months before and after the first month in which the patient presented 5 or more times (the trigger month), a noticeable spike occurred in the trigger month (Figure 2). For the 82 FP5+ patients from the 2012 sample, their total presentations in the 12 months preceding each participant's trigger month accounted for 1,064 ED visits, averaging 13.0 per year or a median of 8 visits, while their total presentations in the 12 months following the trigger month increased by 50% to 1,606 ED visits, averaging 19.6 presentations per year or a median of 15 visits.

With the yearly average presentations by FP5+ patients calculated over a rolling 12-month period across the 2011 to 2016 time frame, ITS analysis revealed that changes in presentation across the time period were –0.854 (95% CI, –1.866 to 0.157), and the trend change was –0.851 (95% CI, –1.722 to 0.021) per year. Neither of these changes was statistically significant. Examination of the residuals of the ITS model, including the autocorrelation plot and the Shapiro-Wilk test of normality (*P*=0.344), did not flag concerns about the analysis used.^25^

## DISCUSSION

Creation of a structured framework for investigating the presentation patterns of people who frequently present to EDs (ie, those with 5 or more ED visits in a calendar month) shows that rolling monthly tracking, rather than annual tracking, can better identify these individuals. The cyclical or chunked patterns revealed in these rolling monthly data show that adopting such an approach may also help prompt timely patient-centered interventions. Recognition of a usual pattern of cyclical high-frequency presentations within a short period (chunked pattern) can avoid misrepresentation of data, help to prevent the clumping of data evident in annual reporting, and enhance detection of changing patient or community needs. Community services, often triggered by activation of an individual patient management plan during an ED presentation, require adequate resources to manage patients in the community and limit re-presentations. Monthly analysis of patient care data may enable more rapid identification and better response to a care consumer's (patient's) possible unmet care needs, identify individual and social determinants that could prompt frequent presentation, and even discriminate between these factors. Alignment of ED presentation frequency with significant anniversaries, days of the week, pension/funding days, or other significant community events could be established and then managed. Triggers for ED presentations are particularly critical for patients who might otherwise come to rely on EDs as a single access point to all care processes.^20^

Yearly figures may not have sufficiently detailed discriminatory capacity to intervene for patients who could benefit the most from an ED-based intervention such as development of a case management plan.^26,27^ Too often, yearly figures include patients for whom acute but transient conditions are present.^5^ Depending on the available staffing resources, 5 presentations in 1 month not only provide a reasonable clinical rationale but also a practical number of presentations to prompt intervention. Many frequent ED presenters have complex issues including mental health illnesses and drug and alcohol addictions.^12,14^ By monitoring presentations monthly (as shown in Figure 1), ED staff can obtain information about changing patterns and frequencies of presentation. Experience suggests that some instances of sudden, frequent ED visits are triggered for many years by an annual event such as the anniversary of the death of a loved one.^20,28^ While without individual case reviews, ED staff will remain unclear about whether successive spikes are associated with the same unsolved complex issues or a different set of problems, future combinations of individual case reviews and analysis of rolling monthly ED presentation data may help preempt and resolve demands on ED services. In the future, as more hospitals move to an integrated electronic medical record, patients who present frequently within a short space of time to multiple EDs (ie, a bout of presentations) might be more easily identified.

Examination of these data also helps to develop and justify the use of segmented regression analysis of ITS studies as an effective research methodology for assessing the requirement for and potential evaluation of interventions for frequent ED presenters.^25^ A monthly frequency count allows an evaluation of an intervention based on the reduced number and trajectory of ED presentations using ITS analysis, rather than the annual approach, which, because of a range of initial patient presentation times, may demand many years of data collection to gather enough data points to demonstrate interventional impacts. Uncontrolled before-and-after studies, such as those commonly used to illustrate the efficacy of some clinical intervention to better manage frequent presenters,^14,26,27,29-32^ may lead to misinterpretation of the impacts of interventions^24^ and thus misdirect future clinical care. As illustrated in Figure 2, the average number of presentations by participants peaked in the first occurrence of ≥5 monthly presentations. These data suggest a change in average presentations would occur before and after this trigger month, even following a placebo intervention. The design of case management interventions for patients who present to EDs frequently over variable periods of time can be complex, reflecting each patient's specific needs. Evaluation of such complex interventions should be matched by evaluation methodology that can diagrammatically and statistically incorporate such complexity. Thus, a methodology underpinned by ITS analysis is likely to be a more efficacious alternative to the traditional annual data accumulation (chunking) that currently occurs.^24,26^

### Limitations

Importantly, this study does not propose a new intervention program, nor does it evaluate existing programs. Rather, this study used an existing baseline dataset of recorded ED presentations by patients identified as FP5+ to demonstrate more accurate and reliable data tracking that better fits the bout-like pattern of presentations these patients often show.

Despite the well-established patient identification policy at the ED study site, data recording errors could have resulted in a few incorrect counts of presentations (frequent or infrequent). Additionally, some care consumers (patients) might have presented at multiple hospitals during the study period, thus distorting their presentation patterns. Care consumers (patients) may have also moved to other health regions, resolved their health issues, or even died.

## CONCLUSION

People with complex care needs who present several times to EDs often present challenges in terms of economics and processes. Despite widespread recognition of these challenges, the tools to systematically explore frequent presenters and report the impacts of interventions remain variable and can lead to misrepresentation of intervention impact. The ≥5 presentations in a calendar month definition used in this study is proposed as an appropriate definition for people to be classed as frequent presenters to EDs in future care allocation and studies. This definition, coupled with a rolling monthly approach to individual ED presentations instead of the 12-month model, may prompt more responsive patient care delivery and management, as well as form the basis for reliable evaluation of interventions in the future.

## Notes

*This article meets the Accreditation Council for Graduate Medical Education and the American Board of Medical Specialties Maintenance of Certification competencies for Patient Care, Medical Knowledge, and Practice-Based Learning and Improvement.*
